# Some Like It Hot: Heat Resistance of *Escherichia coli* in Food

**DOI:** 10.3389/fmicb.2016.01763

**Published:** 2016-11-03

**Authors:** Hui Li, Michael Gänzle

**Affiliations:** ^1^Department of Agricultural, Food and Nutritional Science, University of Alberta, EdmontonAB, Canada; ^2^College of Bioengineering and Food Science, Hubei University of TechnologyHubei, China

**Keywords:** *Escherichia coli*, heat resistance, VTEC, food processing, protein, locus of heat resistance

## Abstract

Heat treatment and cooking are common interventions for reducing the numbers of vegetative cells and eliminating pathogenic microorganisms in food. Current cooking method requires the internal temperature of beef patties to reach 71°C. However, some pathogenic *Escherichia coli* such as the beef isolate *E. coli* AW 1.7 are extremely heat resistant, questioning its inactivation by current heat interventions in beef processing. To optimize the conditions of heat treatment for effective decontaminations of pathogenic *E. coli* strains, sufficient estimations, and explanations are necessary on mechanisms of heat resistance of target strains. The heat resistance of *E. coli* depends on the variability of strains and properties of food formulations including salt and water activity. Heat induces alterations of *E. coli* cells including membrane, cytoplasm, ribosome and DNA, particularly on proteins including protein misfolding and aggregations. Resistant systems of *E. coli* act against these alterations, mainly through gene regulations of heat response including EvgA, heat shock proteins, σ^E^ and σ^S^, to re-fold of misfolded proteins, and achieve antagonism to heat stress. Heat resistance can also be increased by expression of key proteins of membrane and stabilization of membrane fluidity. In addition to the contributions of the outer membrane porin NmpC and overcome of osmotic stress from compatible solutes, the new identified genomic island locus of heat resistant performs a critical role to these highly heat resistant strains. This review aims to provide an overview of current knowledge on heat resistance of *E. coli*, to better understand its related mechanisms and explore more effective applications of heat interventions in food industry.

## Introduction

Pasteurization and domestic cooking are common interventions for reducing the numbers of vegetative bacterial cells including pathogens in food. Heat kills vegetative bacterial cells by inactivation of cellular components, particularly membranes, proteins, and ribosomes (Tsuchido et al., 1985; Mackey et al., 1991; Mohácsi-Farkas et al., 1999; Lee and Kaletunc, 2002). Thermal food processing has an excellent record of establishing and maintaining food safety. However, consumer preferences for raw or minimally processed food, and the aim to minimize thermal degradation of nutrients are incentives to reduce the intensity of thermal processing. Moreover, fresh foods including meats and produce cannot be heated to temperature that are lethal to all pathogens, and bacterial pathogens are highly resistant to thermal processing in the dry state (Santillana Farakos et al., 2014; Syamaladevi et al., 2016). In addition, the heat resistance of pathogens is variable and heat resistant strains may withstand thermal processes that are lethal to the majority of strains of the same species (Ng et al., 1969; Murphy et al., 1999; Dlusskaya et al., 2011).

**FIGURE 1:**
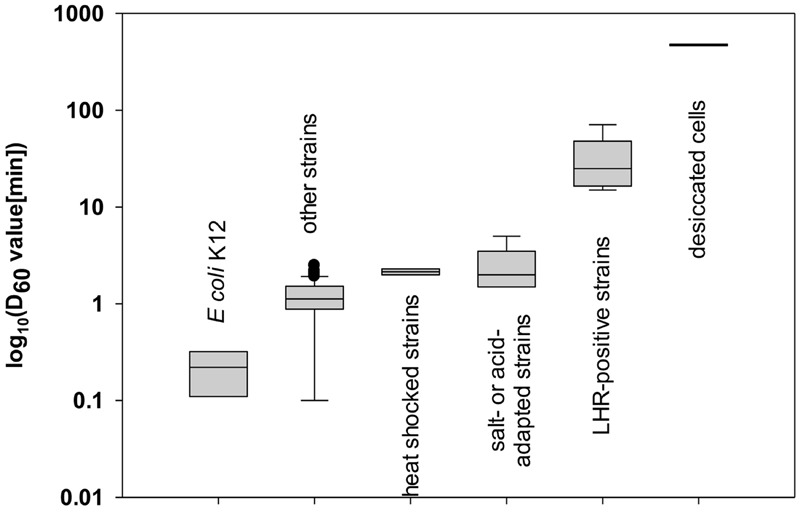
**Heat resistance of *Escherichia coli*.** Data shown are log_10_ value of D_60_ (min) of 144 strains collected from past publications: three values of K-12 strains (Chung et al., 2007; Jin et al., 2008; Dlusskaya et al., 2011), 125 of other strains of *E. coli* (Juneja and Marmer, 1999; Dlusskaya et al., 2011; Enache et al., 2011; Pleitner et al., 2012; Liu, 2015; Mercer et al., 2015), 2 D-values of strains after overexpression of heat shock proteins (HSP) (Hauben et al., 1997; Ruan et al., 2011), 7 D-values of strains after adaptation to salt or acid stress (Buchanan and Edelson, 1999; Pleitner et al., 2012; Garcia-Hernandez et al., 2015), 5 D-values of LHR positive strains (Pleitner et al., 2012; Mercer et al., 2015), and 2 D-values of strains treated by dry heat (Neetoo and Chen, 2011; Kim et al., 2015).

*Escherichia coli* has been considered to be a relatively heat sensitive organism; however, strains of *E. coli* belong to the most heat resistant vegetative foodborne pathogens (**Figure 1**; Jay et al., 2005; Doyle and Beuchat, 2013). Heat resistant *E. coli* have D_60_ value of more than 6 min (**Figure 1**; Liu et al., 2015; Mercer et al., 2015), and their resistance matches or exceeds *Salmonella* Senftenberg 755 with D_60_ of 6.3 min (Ng et al., 1969; Baird-Parker et al., 1970) and *Staphylococcus aureus* with D_60_ of 4.8-6.5 min (Jay et al., 2005; Kennedy et al., 2005; Doyle and Beuchat, 2013). Foodborne disease with *E. coli* has been linked to consumption of meat and meat products as well as fruits and fresh produce (Frenzen et al., 2005; Karch et al., 2005; Greig and Ravel, 2009; Yeni et al., 2015). Heat treatments for effective microbial decontamination and minimum organoleptic deterioration of foods (Woodward et al., 2002; Klaiber et al., 2005; Rajic et al., 2007) necessitate knowledge of the heat resistance of target foodborne pathogens as well as factors influencing heat resistance. This review aims to provide an overview of current knowledge on mechanisms of heat resistance of *E. coli* to provide novel perspectives on conventional and novel thermal processing of foods. Major mechanisms of heat resistance are active in all strains of *E. coli*; however, relatively few studies elucidated genetic determinants for strain-specific acquisition of heat resistance. A recently identified genomic island termed locus of heat resistance (LHR) substantially increases the heat resistance of about 2% of strains of *E. coli* (Mercer et al., 2015). Where appropriate, *E. coli* will be compared to *Salmonella enterica*, a closely related organisms exhibiting comparable resistance to heat.

## Variability of Resistance of Strains of *E. coli* to Heat

The D_60_-value of *E. coli* K12 is reported as 0.1 to 0.3 min (Chung et al., 2007; Jin et al., 2008; Dlusskaya et al., 2011); however, a majority of strains of *E. coli* exhibits D_60_-values exceeding that value up to 10-fold (**Figure 1**). Heat resistance is not related to the phylogenetic group, the serotype, or the virotype of *E. coli* (Liu et al., 2015; Mercer et al., 2015). Highly heat resistant strains of *E. coli* exhibit D_60°C_ values exceeding 10 min (Dlusskaya et al., 2011; Garcia-Hernandez et al., 2015). Genetic determinants of the variability of heat resistance between strains are only partially understood. An overview on isogenic mutant strains of *E. coli* and their heat resistance is shown in **Table 1**. Genes that are related to the heat shock response, including the alternative sigma factors σ^H^ and σ^E^, the heat shock proteins (HSPs) IbpA/B, the alternative sigma factor σ^S^ regulating the general stress response, the oxidative stress response regulated by SodA/B, and genes related to envelope properties including synthase of colanic acid, cyclopropane fatty acids (CFAs), NmpC and EvgA relate to heat resistance (**Table 1** and references therein). *E. coli* strains deficient of in σ^H^, σ^S^, SodA/B, IbpA/B, and colanic acid as well as CFAs were more sensitive to heat compared to their isogenic parental strains. Overexpression of EvgA increased heat resistance (**Table 1**). The LHR (**Table 1**) mediates extreme heat resistance with D_60_-values of 10 min or higher (**Table 1**). The heat resistance of strains of *E. coli* also depends on the food matrix (**Table 2**). The resistance of *E. coli* LTH5807 to heating on mung bean, radish, or alfalfa seeds differed substantially (**Table 2**). The survival of the LHR-positive *E. coli* AW1.7 in beef patties cooked to 71°C provides further evidence that the heat resistance of *E. coli* depends on the food matrix. Heat treatments that are considered to be lethal to *E. coli* thus may fail to safely eliminate contaminating *E. coli* (**Table 2**).

**Table 1:** Effect of gene disruption or overexpression on heat resistance of *E. coli.*

*Escherichia coli* serotype or strain number	Heat conditions (T/time)	Lethality (logN/N_0_)	Medium /products	Reference
MC4100 (parental strain) KY1601 (△*rpoH*)	57°C, 2 min	<0.1 >3.5	M9 medium	Jenkins et al., 1991
AB1157 (parental strain) JI132 (△*sodA sodB* strain)	48°C, 2 h	<0.5 >6	LB broth	Benov and Fridovich, 1995
ATCC 43895 (parental strain) FRIK 816-3 (△*rpoS*)	55°C, 7 min	<1 >4	Fermented sausage	Cheville et al., 1996
MC4100 MC4100 (△*ibpA/B*)	50°C, 4 h	<2 >3	LB broth	Kuczyñska-Wiśnik et al., 2002
W6-13 (parental strain) M4020 (△*wca*)	60°C, 5 min	3.3 6.6	Minimal glucose broth	Mao et al., 2001
AW1.7 AW1.7 (△*cfa*)	60°C, 30 min	2.0 3.1	LB broth	Chen and Gänzle, 2016
MG1655 MG1655 (△*cfa*)	57°C, 15 min	1.3 2.2	LB broth	Chen and Gänzle, 2016
BL21 overexpression of IbpA/IbpB	50°C, 30 min	1.5 0.7-0.9	M9 medium	Kitagawa et al., 2000
*E. coli* W3110 overexpression of EvgA	50°C, 2 h	5 1.5	TY broth	Christ and Chin, 2008
GGG10 overexpression of NmpC	60°C, 1 min	3.5 0.5	LB broth	Ruan et al., 2011
AW1.7 (pRK767) AW1.7 △pHR1 (pRK767) AW1.7 △pHR1 (pLHR)	60°C, 5 min	<1 >8 <1	LBbroth	Mercer et al., 2015

*LB, Luria-Bertani; TY, Tryptone-yeast extract.*

**Table 2:** Examples of heat resistance of *E. coli* strains in food.

*Escherichia coli* serotype or strain number	Heat conditions (*T*/time)	Lethality (logN_0_/N)	Medium /products	Reference
LTH5807 (O157:H^-^; stx^-^)	60°C, 10 min 60°C, 3 min 60°C, 4 min	>7.2 >7.2 5.9	Mung bean Radish Alfalfa	Weiss and Hammes, 2005
204P (O157:H7)	50°C, 300 min 55°C, 30 min	3-5 2-4	Pork sausage (7-30% fat)	Ahmed et al., 1995
AW1.7 AW1.7 △pHR1 GGG10	Internal 63/71°C	3-5^#^/3.5 4-7^#^/5 4.5/UDL	Beef patties	Liu et al., 2015
MG1655 (K12), LMM1030	Internal 63°C	5-6^#^	Beef patties	Liu et al., 2015
O26, O104, O111, O121, and O157	Internal 63°C	2-NC	Beef patties	Liu et al., 2015
O26, O104, and O121	Internal 71°C	6-NC	Beef patties	Liu et al., 2015
O157:H7 (VTEC) Non-O157 (VTEC)	Internal 49-71°C	3.2-4.1 2.5-4.5	Beef steaksˆ	Luchansky et al., 2012
8- strain VTEC cocktail^∗∗^	191.5°C, ≤ 1.25 min 1.5-2.5 min	1.6-5.1 UDL	Single cubed Beef steaks	Swartz et al., 2015
8- strain VTEC cocktail^∗∗^	≤3.0 min 3.5 min	0.8-5.3 UDL	Double cubed Beef steaks	Swartz et al., 2015

	**Temperature**	***D* value (min)**		

O157:H7 E0139 SEA 13B88	57°C	8.2/9.1 6.2/7.9	Cantaloupe/wat- ermelon juice	Sharma et al., 2005
Heat resistant strains of 7 VTEC serotypes (O26, O45, O103, O111, O121, O145, and O157)	56°C 60°C 62°C	2.1-4.5 0.4-1.0 0.2-0.5	Apple juice	Enache et al., 2011
ATCC25922	55°C	10.9	Goat milk	Pereira et al., 2006
380-94 (O157:H7)	58°C 60°C 62°C	14.4 6.1 2.5	Postfermented pepperoni	Riordan et al., 2000
4-strains cocktail of EDL-931, A 9218-C1, 45753-35, 933 (all are O157:H7)	55°C 60°C 65°C	11.5-12.0 1.9-2.0 0.3-0.4	Ground turkey, lamb, and pork	Juneja and Marmer, 1999

*UDL, cell counts after treatment were under detection limit.*

*NC, no surviving cells after enrichment.*

*#Reductions depend on fat content from 15 to 35% in ground beef.*

*ˆThickness of beef steaks is 2.54 or 3.81 cm; initial cell counts are around 5.50 cfu/g.*

*^∗∗^Temperature is the surface temperature; cooking time refers to the time per side; initial cell counts are around 6.3-6.8 cfu/g.*

## Mechanisms Related to Outer Membrane and Membrane Fluidity

**FIGURE 2:**
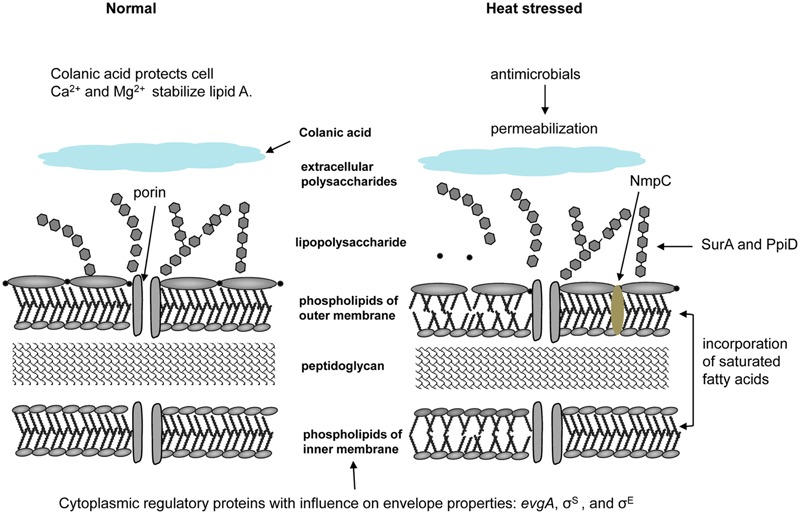
**Heat effects on cell membranes and attributes to heat resistance of *E. coli*.** Extracellular polysaccharides including colanic acid forms a thick mucoid matrix on cell surfaces and provide protection of cells; disruption of *wsc* genes required for colanic acid biosynthesis substantially decreased heat resistance when compared to its parental strain (Whitfield and Valvano, 1993; Mao et al., 2001). LPS is a barrier to prevent rapid penetration of hydrophobic molecules, and is stabilized by divalent cations Mg^2+^ and Ca^2+^ against heat or pressure stress (Hitchener and Egan, 1977; Vaara, 1992; Hauben et al., 1998; Li et al., 2016). The solute transport proteins and the outer membrane porin NmpC contribute to heat resistance of *E. coli* AW1.7 (Ruan et al., 2011). Addition of antimicrobials including chitosan decreased the heat resistance due to the increased permeability of outer membrane (Liu, 2015). The master transcriptional regulator *evgA* is a cytoplasmic protein that increased heat resistance through activation of genes involved in periplasmic functions (Christ and Chin, 2008). The alternative sigma factors σ^S^ and σ^E^ also influence the properties of cell envelope (Lange and Hengge-Aronis, 1991; Bukau, 1993). LPS proteins SurA and PpiD lead to overall reduction in the level and folding of outer membrane proteins, consequently induce the periplamic heat shock response (Missiakas et al., 1996; Dartigalongue and Raina, 1998). Incorporating more saturated fatty acids such as palmitic acid and cyclopropane fatty acids (CFAs) into membrane lipids antagonizes the heat-induced increase in fluidity and achieves an ideal physical state of membrane (Katsui et al., 1981; Ruan et al., 2011; Chen and Gänzle, 2016). Disruption of *cfa* coding for CFA synthase of *E. coli* AW1.7 and MG1655 induced accumulation of the unsaturated fatty acid C16:1 and C18:1 in membrane lipids, consequently reducing the heat resistance of them (Chen and Gänzle, 2016).

Cell surface structures and appendages provide the first line of defense to environmental stress. An overview of heat stress responses related to cell membranes and the periplasm is provided in **Figure 2**. Most strains of *E. coli* secrete extracellular polysaccharides, including colanic acid, which forms a thick mucoid matrix on the cell surface (Whitfield and Valvano, 1993; Mao et al., 2001). A colanic acid-deficient mutant of *E. coli* M4020, obtained by insertional disruption of the *wsc* genes required for colanic acid biosynthesis, was less tolerant to exposure to 55 and 60°C than its parental strain *E. coli* O157:H7 W6-13 (**Table 1**), indicating that colanic acid confers heat resistance to *E. coli* O157:H7 (**Figure 2**) (Mao et al., 2001). Lipopolysaccharide (LPS) serves as a barrier to prevent rapid penetration of hydrophobic molecules, and is stabilized by divalent cations, particularly Mg^2+^ and Ca^2+^ (**Figure 2**) (Hitchener and Egan, 1977; Vaara, 1992; Hauben et al., 1998; Li et al., 2016). Expression of the outer membrane porin NmpC increased survival of *E. coli* GGG10 at 60°C by 50- to 1,000-fold (**Figure 2**) (Ruan et al., 2011). The outer membrane permeabilizing polysaccharide chitosan decreased the heat resistance of *E. coli* in apple juice at 60°C (Liu, 2015). The pronounced effect on heat resistance of chitosan occurred on EHEC when combined with rutin or resveratrol in beef patties, due to the greater bacterial destruction from outer membrane to cytoplasmic membrane (Nair et al., 2016).

The fluidity of the membrane influences its function (Zhang and Rock, 2008). The adjustment of membrane lipid composition and membrane fluidity by homoviscous adaptation is a major contributor to the bacterial resistance to heat stress (Sinensky, 1974; Arneborg et al., 1993; Denich et al., 2003; Yuk and Marshall, 2003; Yoon et al., 2015). Adaptive systems responding to heat stress in *E. coli* contribute to the stabilization of membrane-bound enzymes, and affect physical properties of the cytoplasmic membrane (Torok et al., 1997; Beney and Gervais, 2001). Remarkably, heat resistance induced by slow heating of *E. coli* was related to adaptation of the membrane fluidity rather than protein synthesis (Guyot et al., 2010). Heat-adaptation increased the heat resistance of *E. coli* strains by the maintenance of the membrane in the liquid-crystalline state. The incorporation of saturated fatty acids into membrane lipids reduces membrane fluidity (Nakayama et al., 1980; Katsui et al., 1981) and consequently antagonizes the heat-induced increase in fluidity (**Figure 2**) (Quinn, 1981; De Mendoza and Cronan, 1983; Suutari and Laakso, 1994; Mejía et al., 1995; Yuk and Marshall, 2003). The heat resistant *E. coli* AW1.7 was characterized by a higher proportion of saturated and CFAs in the cytoplasmic membrane when compared to heat sensitive strains of *E. coli* (**Figure 2**) (Ruan et al., 2011). A contribution of CFAs to heat resistance of *E. coli* was confirmed by disruption of *cfa* coding for CFA synthase (Chen and Gänzle, 2016). The *cfa* deficient derivatives of *E. coli* AW1.7 and MG1655 did not produce CFAs; the unsaturated fatty acid C16:1 and C18:1 replaced CFAs in membrane lipids and the mutant strain was less resistant to heat when compared to the parent strains (**Figure 2**) (Chen and Gänzle, 2016).

## Regulation of Heat Response By EvgA, HSPs, and σ^E^

**FIGURE 3:**
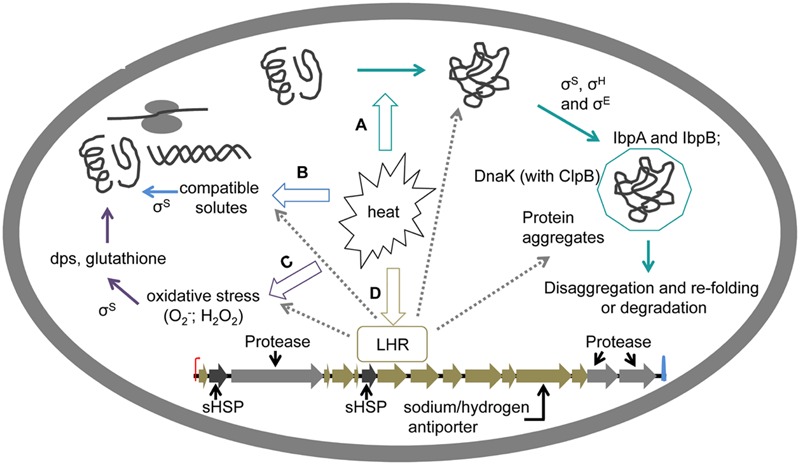
**Cytoplasmic determinants of heat resistance in *E. coli*. (A)** Preventions of protein aggregation. Heat enhances misfolding of proteins and consequently induces protein aggregation. General stress response factors σ^S^, σ^H^, and σ^E^, as well as some small HSPs can suppress protein aggregation (Parsell and Lindquist, 1993; Landini et al., 2014). Small HSPs IbpA and IbpB bind to misfolded proteins and thus contribute to disaggregation of during sublethal heat shock (Laskowska et al., 1996; Veinger et al., 1998; Kuczyñska-Wiśnik et al., 2002). The DnaK system acts together with ClpB to prevent protein aggregation induced by heat (Mogk et al., 1999, 2003). **(B)** Compatible solutes accumulation induced by salt contributes to heat resistance through overcoming osmotic stress and stabilizing ribosomes (Ramos et al., 1997; Lamosa et al., 2000; Pleitner et al., 2012). Accumulation of amino acids including glycine betaine and proline as major cytoplasmic solutes, and the accumulation of carbohydrates including glucose and trehalose occurred in response to the addition of NaCl in *E. coli*, resulting in increased thermal stability of ribosomes during heat treatment (Pleitner et al., 2012). Mannosylglycerate and diglyerol phosphate protect proteins during heat treatment (Ramos et al., 1997; Lamosa et al., 2000). **(C)** Mitigation of oxidative stress. Oxidative stress induced by heat damages intracellular components including proteins, ribosomes and DNA. The general stress response factor σ^S^ and the DNA binding protein dps acts against oxidative stress (Choi et al., 2000; Zhao et al., 2002; Landini et al., 2014). Pyruvate and catalase contribute to recovery of sublethally injured cells after heat treatments (Czechowicz et al., 1996; Mizunoe et al., 2000). **(D)** Regulation of the locus of heat resistance (LHR). LHR is unique genomic island contributing to extreme heat resistance in *E. coli* (Mercer et al., 2015). LHR contains 16 predicted ORF encoding small HSPs (sHSP, Orf2, and Orf7), hypothetical proteins yfdX family (Orf8 and Orf9), proteases (Orf3, Orf15, and Orf16), thioredoxin (Orf12), and sodium/hydrogen antiporters (Orf13), accordingly contributing to heat shock response, osmotic stress response, turnover of misfolded or disaggregation proteins, oxidative stress response, osmotic and heat stress response, respectively (Mercer et al., 2015; Lee et al., 2016). Predicted functions of LHR are indicated by dashed lines.

Cytoplasmic mechanisms of heat resistance relate to the effect of HSPs and compatible solutes on protein folding, and to oxidative stress (**Figure 3**). The regulation of the heat shock response of *E. coli* is governed by the two alternative sigma factors σ^H^ and σ^E^ (**Figure 3A**). The heat shock response is induced by temperatures around the growth/no-growth interface which aggravate protein misfolding but permit gene expression and protein synthesis (Lindner et al., 2008; Winkler et al., 2010; Govers et al., 2014; Lee et al., 2016). σ^H^ and σ^E^ are encoded by *rpoH* and *rpoE*, regulate transcription of heat-shock regulons coping with protein misfolding in the cytoplasm and the periplasm, respectively, and mediate cytoplasmic stress and envelope stress responses (Bukau, 1993). HSPs including chaperones and proteases function by holding partially unfolded proteins to prevent aggregation of heat-denatured proteins, and disaggregation of denatured proteins to allow refolding or proteolytic degradation (Parsell and Lindquist, 1993; Landini et al., 2014; Lee et al., 2016). The small HSPs IbpA and IbpB are holdases; DnaK, DnaJ, GrpE facilitate protein folding during translation, and guide aggregated proteins to the disaggregase ClpB. ClpP and other heat-shock proteases degrade aggregated proteins. The expression of HSPs is induced by σ^H^ under sublethal heat stress and increases heat resistance of *E. coli* (Arsène et al., 2000). A σ^H^ deletion in *E. coli* eliminated synthesis of HSPs including DnaK, GroEL, and HtpG and the resulting strain was very sensitive to exposure to 57°C (**Table 1**). Starvation significantly enhanced the heat resistance of this strain (Jenkins et al., 1991). Small HSPs prevent protein aggregation by heat (Jakob et al., 1993; Lee et al., 1997; Kitagawa et al., 2000; Mogk et al., 2003). Overexpression of IbpA and IbpB increased resistance not only to heat but also to superoxide (Kitagawa et al., 2000; **Table 1**). Small HSPs IbpA and IbpB prevent the aggregation of denatured endogenous proteins (Laskowska et al., 1996; Veinger et al., 1998; Kuczyñska-Wiśnik et al., 2002). The DnaK system also prevented protein aggregation induced by heat. This disaggregation is more efficient when DnaK acts in concert with ClpB (Mogk et al., 1999, 2003). However, disruption of *clpA, htpG*, and *ibp* in *E. coli* did not affect the viability at 50°C (Thomas and Baneyx, 1998). The pressure resistant strains *E. coli* LMM1010, LMM1020, and LMM 1030 exhibit an increased basal expression of HSPs including DnaK, Lon, and ClpX; this increased expression may also account for the moderate increase of heat resistance of these strains (Hauben et al., 1997; Aertsen et al., 2004). Overall, the inducible heat shock response is a key contributor for growth of *E. coli* at temperature exceeding the optimum temperature of growth, but it makes only a modest contribution to the strain-specific differences of the resistance to lethal heat challenge.

Four key proteins involve in the regulation of σ^E^-dependent envelope stress response, including RseA, RseB, DegS, and Yael (Alba and Gross, 2004). The activity of σ^E^ is modulated by the expression of outer membrane proteins and outer membrane proteins induce σ^E^ activity (Mecsas et al., 1993). Moreover, deletions of LPS proteins SurA and PpiD lead to overall reduction in the level and folding of outer membrane proteins, and to the induction of the periplamic heat shock response (**Figure 2**) (Missiakas et al., 1996; Dartigalongue and Raina, 1998).

A master transcriptional regulator *evgA* activates genes involved in periplasmic functions, as well as in membrane and permeability functions. Its overexpression significantly increases heat resistance of *E. coli* (Christ and Chin, 2008; **Table 1**; **Figure 2**). The response regulator EvgA is part of a two-component regulatory system with sensor kinase EvgS, binding the intergenic region of *evgAS* and *emrKY* coding for eﬄux pump, and regulating the expression of both operons (Kato et al., 2000). Comparison of the genome-wide transcription profile of EvgA-overexpressing and EvgA-lacking strains revealed that EvgA conferred acid resistance to *E. coli* (Masuda and Church, 2002). EvgA controls the expression of wide range of genes, including *gadABC, hdeAB, emrKY, yhiUV*, and *yfdX* which are related to acid resistance, osmotic adaptation, drug resistance and other functions (Nishino et al., 2003).

## Regulation of Heat Resistance By σ^S^, and Cross-Resistance to Acid, Oxidative, and High Pressure Stress

Stationary phase cells are more resistant than exponential phase cells, mainly because of the increased expression of σ^S^ (**Figure 3A**) (Cheville et al., 1996; Kaur et al., 1998). The σ^S^ regulon contributes to the general stress response and increase acid, heat, and / or osmotic resistance of *E. coli* (Hengge-Aronis et al., 1991; Cheville et al., 1996; Robey et al., 2001; Hengge-Aronis, 2002; Allen et al., 2008; Landini et al., 2014). Adaptation to acid stress provides cross-protection to heat stress (Ryu and Beuchat, 1998; Buchanan and Edelson, 1999; Ryu and Beuchat, 1999; Mazzotta, 2001; Yuk and Marshall, 2003). For example, adaptation of enterohemorrhagic *E. coli* to pH 4.6 increased the heat resistance at 58°C 2-4 fold when compared to cells grown at pH 7.0 (Buchanan and Edelson, 1999). Induction of acid resistance in *E. coli* O157:H7 increases levels of CFAs in the cytoplasmic membrane (Brown et al., 1997), which stabilize cells against several environmental stressors including heat (Grogan and Cronan, 1997; Chen and Gänzle, 2016). Moreover, σ^S^ dependent gene expression increased the heat resistance of *E. coli* O157:H7 after adaptation to temperatures above the optimum growth temperature (Cheville et al., 1996; Yuk and Marshall, 2003; **Table 1**). Starvation of *E. coli* O157:H7 substantially increased D_52_-values; this enhanced heat resistance was related to the expression of starvation-induced proteins UspA and GrpE (Zhang and Griffiths, 2003).

Heat induces production of O_2_ in *E. coli* under aerobic conditions, possibly by disruption of the electron transport systems of the membrane, and consequently induces the manganese-containing superoxide dismutase (Privalle and Fridovich, 1987). Accumulation of reactive oxygen species after exposure to sublethal stress results in lethal damage to DNA, RNA, proteins, and lipids (Aldsworth et al., 1999; Cabiscol et al., 2000; Aertsen et al., 2005). The general stress response factor σ^S^ also protects against oxidative stress (**Figure 3C**) (Landini et al., 2014). The σ^S^-regulated DNA binding protein dps binds DNA as homo-dodecamer and prevents DNA damage by oxidative stress or low pH (Choi et al., 2000; Zhao et al., 2002). The synthesis of CFAs in *E. coli* also increases resistance to oxidative stress (Grogan and Cronan, 1997). Proteins that are alter the resistance of *E. coli* to pressure-induced oxidative stress, including systems for thiol-disulfide redox homeostasis and proteins containing iron-sulfur clusters, probably also contribute against oxidative stress induced by heat (Malone et al., 2006; Charoenwong et al., 2011; Imlay, 2013; Gänzle and Liu, 2015).

Oxidative stress induced by sublethal thermal damage may also account for the phenomenon termed “viable but nonculturable state” (VBNC). VBNC cells cannot be detected by standard culture techniques but can be resuscitated under favorable conditions (Bogosian et al., 2000; Gupte et al., 2003; Morishige et al., 2013). Addition of sodium pyruvate recovered cells of *E coli* after heat-induced sublethal injury. This protective effect was related to the ability of pyruvate to degrade hydrogen peroxide (Czechowicz et al., 1996; Mizunoe et al., 2000). Addition of sodium pyruvate or catalase to medium agar also resuscitated VBNC *Salmonella* Enteritidis or *Vibrio vulnificus* cells, respectively, which had become sensitive to hydrogen peroxide (Bogosian et al., 2000; Morishige et al., 2013).

## Effects of Salt or Sugar Addition in High Moisture Foods

The water activity of food and particularly the salt content influence the heat resistance of *E. coli*. *E. coli* responds to an increase of the osmotic pressure by accumulation or synthesis of compatible solutes, small organic solutes that balance the osmotic pressure without interfering with cytoplasmic functions (Kempf and Bremmer, 1998). High cytoplasmic concentrations of compatible solutes increase heat resistance of *E. coli* and other bacterial cells by stabilizing ribosomes and proteins through a mechanisms referred to as “preferential hydration” (**Figure 3B**) (Ramos et al., 1997; Lamosa et al., 2000; Pleitner et al., 2012). A reduction in water activity from 0.995 to levels between 0.98 and 0.96 in salt or sucrose solutions significantly enhanced the heat resistance of *E. coli* (Kaur et al., 1998). The heat resistance of several strains of *E. coli* was also increased by addition of 2–6% of NaCl (Garcia-Hernandez et al., 2015). Addition of 2% NaCl resulted in the accumulation of amino acids including glycine betaine and proline as major cytoplasmic solutes; accumulation of carbohydrates including glucose and trehalose occurred in response to the addition of 6% NaCl (Pleitner et al., 2012). The accumulation of solutes corresponded to an increased heat resistance of *E. coli*, and a higher thermal stability of ribosomes (Pleitner et al., 2012). The effect of NaCl addition on solute accumulation and heat resistance of *E. coli* is observed at concentrations that are typical for food systems. A critical concentration of NaCl in ground beef, about 2.7-4.7%, substantially increased heat resistance of *E. coli* O157:H7 at 55-62.5°C (Juneja et al., 2015). In addition, pre-exposure to 5% NaCl at room temperature for 24 h increased the heat resistance of *E. coli* O157:H7 at 55°C (Bae and Lee, 2010).

The effect of the fat content on heat resistance of *E. coli* is controversial. An increased fat content in food products increased the heat resistance of *E. coli* in some studies (Line et al., 1991; Huang et al., 1992; Ahmed et al., 1995; Smith et al., 2001; Liu et al., 2015), while other studies reported decreased resistance, no effect, or strain-specific effects (Kotrola and Conner, 1997; Vasan et al., 2014; Liu et al., 2015). The potential direct effects of fat on heat resistance of *E. coli* are confounded by the strong effect of fat on heat transfer in solid foods. Reduced heat transfer increases the heating times to a certain target temperature and thus profoundly affects process lethality.

## LHR and Extreme Resistance to Heat

Extreme heat resistance of *E. coli* is conferred by the LHR (**Figure 3D**, Mercer et al., 2015). The LHR is a genomic island of about 14 kbp which encodes for 16 genes; six of these genes are unique to heat resistant strains of *E. coli* (Mercer et al., 2015). Acquisition of the LHR increases survival after exposure to 60°C for 5 min by more than 7 log(cfu/mL); the LHR is thus one of the most powerful mediators of heat resistance in *E. coli* (**Table 1**; Mercer et al., 2015). Loss of the LHR also reduces the pressure resistance in *E. coli* AW1.7 (Garcia-Hernandez et al., 2015; Liu et al., 2015; Mercer et al., 2015). Remarkably, the presence of a truncated LHR in wild type strains of *E. coli*, or cloning of fragments of the LHR had little effect on heat resistance, indicating that the 16 genes act in concert to provide heat resistance in LHR-positive strains (Mercer et al., 2015). A genomic island with high similarity to the LHR, the *Pseudomonas aeruginosa* clone C-specific genomic island (PACGI-1) was characterized in *Pseudomonas* (Lee et al., 2015).

The 16 predicted open reading frames (ORF) within LHR encode small HSPs (Orf2 and Orf7), proteins of the YfdX family with unknown function (Orf8 and Orf9), heat shock proteases (Orf3, Orf15 and Orf16), thioredoxin (Orf12), and a sodium/hydrogen antiporter (Orf13) (Mercer et al., 2015). According to the predicted function of proteins encoded by the LHR, the genomic island may thus contribute to the turnover of misfolded or aggregated proteins, the osmotic stress response, and mitigate oxidative stress (Mercer et al., 2015). The contribution of genes encoded by the LHR to protein folding and protein turnover was confirmed in the homologous gene cluster PACGI-1 in *P. aeruginosa* (Lee et al., 2015). The small HSPs sHsp20c and ClpG_GI_ contribute to thermotolerance in *P. aeruginosa* through their function as holdases and disaggregating chaperones (Lee et al., 2015, 2016). Cloning of the homologous LHR proteins in *E. coli*, however, had no influence on the heat resistance in *E. coli* (Mercer et al., 2015), demonstrating that the effect of LHR-encoded genes is species specific, and that extreme heat resistance in *E. coli* necessitates HSPs acting in concert with other biochemical functions.

## Heat Resistance of Desiccated *E. coli*

Desiccated strains of *E. coli* and *Salmonella* are characterized by extreme resistance to physical and chemical stressors including heat (Beuchat and Scouten, 2002; Beuchat et al., 2013; Studer et al., 2013; Syamaladevi et al., 2016). Parameters for the heat inactivation of dry bacterial cells are comparable to the moist heat inactivation of bacterial endospores spores rather than pasteurization (Brandl et al., 2008; Du et al., 2010; Podolak et al., 2010). Hot air roasting of almonds even at very high temperature (130-150 °C) achieve less than a 4 log (cfu/g) reduction of *Salmonella* on almonds (Yang et al., 2010). Similarly a 2 log (cfu/g) reduction of Salmonella on dry alfalfa seeds required 10 days of treatment at 60°C; an equivalent bactericidal effect was achieved after 5 min of treatment with wet heat at 60°C (Jaquette et al., 1996; Neetoo and Chen, 2011).

Mechanisms of dry heat resistance are best understood for *Salmonella* (Podolak et al., 2010; Finn et al., 2013). The heat resistance of *Salmonella* at 75°C in meat and bone meal was higher at a_W_ 0.77 than at a_W_ 0.88 (Riemann, 1968). Comparable to the effect of NaCl in high-moisture foods, the heat resistance of dry cells is related to the intracellular concentration of compatible solutes, including K^+^, glutamate and trehalose. The up-regulation of σ^S^, σ^E^, fatty acid catabolism, and formations of Fe-S clusters and filaments also contribute to the resistance to dry conditions (Finn et al., 2013). It was speculated that the extent and strength of the vibration of water molecules in dry bacteria are limited substantially because of the very low water contents. The low water content thus prevents denaturation of cytoplasmic and membrane proteins even at very high temperatures (Earnshaw et al., 1995; Archer et al., 1998). This mechanism was proposed in analogy to bacterial endospores, where the reduced core water reduces the amount of water associated with proteins, thus preventing thermal denaturation (Nicholson et al., 2000). Desiccation of bacterial cells may also stabilize ribosomal units (Syamaladevi et al., 2016).

Several studies demonstrate that concepts and mechanisms that were identified in *Salmonella* are also relevant in *E. coli*. Desiccated VTEC survived at 70°C for 5 h, thus exhibiting almost the same level of heat resistance as *Salmonella* (Hiramatsu et al., 2005). The lethality of treatments of radish seeds at 60°C against *E. coli* O157:H7 increased as the a_W_ increased from 0.25 to 0.65 and 1.0 (Kim et al., 2015). However, information on the dry heat resistance of *E. coli* remains limited when compared to the information on the wet heat resistance of the organisms.

## Conclusion

The resistance of *E. coli* strains to heat intervention treatments has been widely evaluated in the past decades, particularly using strains of *E. coli* O157: H7. Although *E. coli* has been considered as a relatively heat sensitive organisms, the D_60_- values of some strains of *E. coli* are increased to several minutes or even hours by the heat shock response, adaptation to salt or acid stress, acquisition of the LHR, or desiccation (**Figure 1**). About 2% of *E. coli* including food isolates and pathogens harbor the LHR and exhibit extreme resistance to wet heat (Mercer et al., 2015). The biochemical function of the LHR links to proteins aggregation and folding as well as thiol- and ion homeostasis, however, the mechanisms of LHR –mediated heat resistance are only partially understood. Current pathogen intervention methods or cooking recommendations may not suffice to control these highly heat resistant strains of *E. coli* (Dlusskaya et al., 2011; Liu et al., 2015; Mercer et al., 2015). Additional hurdles need therefore to be developed to assure the inactivation of highly heat resistant strains. Further evaluations on inactivation of heat resistant strains under improved heat interventions and mechanisms of heat resistance allow us to design more effective applications in food industry.

## Author Contributions

All authors listed, have made substantial, direct and intellectual contribution to the work, and approved it for publication.

## Conflict of Interest Statement

The authors declare that the research was conducted in the absence of any commercial or financial relationships that could be construed as a potential conflict of interest.
